# Availability and service provision of multidisciplinary diabetes foot units in Australia: a cross-sectional survey

**DOI:** 10.1186/s13047-021-00471-x

**Published:** 2021-04-07

**Authors:** Uyen Giao Vo, Molly Gilfillan, Emma Jane Hamilton, Laurens Manning, Bijit Munshi, Jonathan Hiew, Paul Edward Norman, Jens Carsten Ritter

**Affiliations:** 1 Vascular Surgery Department, Fiona Stanley Hospital, 11 Robin Warren Drive, Murdoch, Western Australia 6150 Australia; 2 Department of Endocrinology, Fiona Stanley Hospital, 11 Robin Warren Drive, Murdoch, Western Australia 6150 Australia; 3 Multidisciplinary Diabetes Foot Unit, Fiona Stanley Hospital, 11 Robin Warren Drive, Murdoch, Western Australia 6150 Australia; 4 Department of Infectious Disease, Fiona Stanley Hospital, 11 Robin Warren Drive, Murdoch, Western Australia 6150 Australia; 5 School of Medicine, University of Western Australia, 35 Stirling Highway, Crawley, Western Australia 6009 Australia; 6 Department of Podiatry, Fiona Stanley Hospital, 11 Robin Warren Drive, Murdoch, Western Australia 6150 Australia; 7 School of Medicine, Curtin University, Kent Street, Bentley, Western Australia 6102 Australia

**Keywords:** Diabetic foot, Multidisciplinary, Survey

## Abstract

**Background:**

With growing global prevalence of diabetes mellitus, diabetes-related foot disease (DFD) is contributing significantly to disease burden. As more healthcare resources are being dedicated to the management of DFD, service design and delivery is being scrutinised. Through a national survey, this study aimed to investigate the current characteristics of services which treat patients with DFD in Australia.

**Methods:**

An online survey was distributed to all 195 Australian members of the Australian and New Zealand Society for Vascular Surgery investigating aspects of DFD management in each member’s institution.

**Results:**

From the survey, 52 responses were received (26.7%). A multidisciplinary diabetes foot unit (MDFU) was available in more than half of respondent’s institutions, most of which were tertiary hospitals. The common components of MDFU were identified as podiatrists, endocrinologists, vascular surgeons and infectious disease physicians. Many respondents identified vascular surgery as being the primary admitting specialty for DFD patients that require hospitalisation (33/52, 63.5%). This finding was consistent even in centres with MDFU clinics. Less than one third of MDFUs had independent admission rights.

**Conclusions:**

The present study suggests that many tertiary centres in Australia provide their diabetic foot service in a multidisciplinary environment however their composition and function remain heterogeneous. These findings provide an opportunity to evaluate current practice and, to initiate strategies aimed to improve outcomes of patients with DFD.

**Supplementary Information:**

The online version contains supplementary material available at 10.1186/s13047-021-00471-x.

## Background

Diabetes is recognised as the world’s fastest growing chronic condition, with an estimated global prevalence of 422 million [1]. It is among the top ten causes of death in adults globally, with approximately four million deaths worldwide in 2017 [2]. Diabetes-related foot disease (DFD), which affects approximately 6% of the world population [3], contributes significantly to individual patient morbidity and mortality, and impacts heavily upon the wider public health system. DFD manifests as ulcers, infection, and Charcot foot in the presence of peripheral neuropathy and/ or peripheral arterial disease [4]. Compared to those without diabetes, patients with diabetes are ten times more likely to require an amputation [5]. It is estimated that the annual costs of DFD to the Australian health system is approximately AUD 1.6 billion [6]. The economic costs and mortality rates exceed that of many common cancers [7]; the 5-year mortality rate of patients with diabetic foot infections is approximately 50% [8].

Over the past 20 years, evidence has accumulated in support of the multidisciplinary care model for prevention and management of diabetes-related foot complications; and use of multidisciplinary teams in managing DFD is well recognised as standard of care [9, 10]. Whilst there are regularly updated guidelines on best management of DFD, particularly the guidance documents published by the global peak body for diabetic foot disease, the International Working Group on the Diabetic Foot (IWGDF) [4, 11], there is no universally accepted guideline to define the ideal composition of a multidisciplinary team for the management of DFD [12–14]. Indeed, a review of eight national diabetic foot disease guidelines in The Western Pacific region, including Australia, emphasised limited similarity to recommendations made by IWGDF [15]. In Australia, although the National Health and Medical Research Council (NHMRC) produced guidelines which identify a need for improved multidisciplinary care planning, these documents did not provide any specific detail on the inpatient team composition [16]. Being valid for only 5 years after its publication in 2011, there have been no current updates or revisions by the NHMRC. However, independent organisations have continued to publish recommendations. Recently a national accreditation standard for high risk diabetic foot centres was introduced by the National Association of Diabetes Centres [17].

Furthermore, Australia has the highest incidence of major limb amputations across the Western World [18]. Although these numbers are not diabetes-specific, previous research has shown that 50% of all amputees have diabetes [19].

These sobering statistics suggest that the provision of dedicated multidisciplinary diabetes foot units (MDFU) across the country is heterogeneous with major gaps in service provision in some areas.

By conducting a survey among Australian vascular surgeons, the authors aimed to determine the current level of service provision for DFD across Australia and to describe multidisciplinary team composition and function in inpatient and outpatient settings.

## Methods

### Survey design

A twenty-part survey was designed, comprising of multiple choice and opt-in free-text responses regarding the management of patients with DFD in each respondent’s institution. Vascular surgeons were identified as the primary target group for such a survey as they are an integral part of a functioning MDFU and in many hospitals people with DFD complications are admitted under the care of vascular surgery. Questions were directed towards establishing hospital size and patient volume, the specialties responsible for admitting patients with DFD, and availability of a dedicated MDFU. The questionnaire also investigated MDFU’s access to different specialists and whether those specialists had regular sessions in inpatient and outpatient contexts. The complete survey is attached in Additional file 1.

### Survey distribution

Non-random (purposive) technique was used to distribute the online survey. In 2017, the online survey was emailed to all 195 Australian members of the Australian and New Zealand Society for Vascular Surgery. The survey remained open for a period of three months. A single reminder email was sent out six weeks after the initial email. Participants were advised that the survey was voluntary and anonymous.

### Statistical analysis

Returned data were collated and analysed using Microsoft Excel (Microsoft Corporation, Washington, USA). Descriptive statistics were used to display variable data, with numbers and proportions used for categorical data, unless otherwise indicated.

Survey responses with more than two incomplete items were deemed ineligible. As not all survey respondents answered every question, the number of respondents answering a question was used as the denominator for the relevant results of that question.

## Results

### Baseline characteristics of respondents

Responses were received from 52 surgeons (26.7%). Thirty-five vascular surgeons identified themselves as working in tertiary metropolitan hospitals (35/51, 68.6%). Five respondents worked in private sector (5/51, 9.8%) (Table 1).

**Table 1:** Characteristics of the survey’s respondents

State		
	Australian Capital Territory	2/52 (3.8%)
	New South Wales	22/52 (42.3%)
	Northern Territory	0/52 (0%)
	Queensland	7/52 (13.5%)
	South Australia	1/52 (1.9%)
	Tasmania	2/52 (3.9%)
	Victoria	12/52 (23.1%)
	Western Australia	6/52 (11.5%)
**Primary location of practice**	Tertiary metropolitan hospital	35/51 (68.6%)
	Secondary metropolitan hospital	3/51 (5.9%)
	Regional/rural hospital	8/51 (15.7%)
	Private metropolitan hospital	4/51 (7.8%)
	Private regional/rural hospital	1/51 (2.0%)
**Annual DFD inpatient caseload**	< 20	0/51 (0%)
	21–50	7/51 (13.7%)
	51–100	11/51 (21.6%)
	> 100	33/51 (64.7%)
**Annual DFD outpatient caseload**	< 20	1/50 (2.0%)
	21–50	7/50 (14.0%)
	51–100	4/50 (8.0%)
	> 100	38/50 (76.0%)

*DFD* Diabetes-related foot disease

The majority of respondents saw more than 100 DFD patients per year as inpatients (33/51, 64.7%) and outpatients (38/50, 76.0%). Of 33 surgeons seeing more than 100 DFD inpatients per year, 25 of them (75.8%) worked in metropolitan hospitals. Similarly, 79.0% (30/38) of those who saw more than 100 DFD outpatients per year worked in metropolitan hospitals.

### Multidisciplinary diabetes foot unit’s activities

An overview of MDFU services is displayed in Table 2. Only 59.6% of respondents reported availability of MDFU in their institutions (31/52). Most of these institutions were identified as tertiary metropolitan hospitals (26/31, 83.9%), with four respondents working in regional/rural hospitals and one in a secondary metropolitan hospital. None of the private hospitals captured in this survey had a multidisciplinary service for patients with DFD.

**Table 2:** Availability of and services provided by Multidisciplinary Diabetic Foot Unit

Institution with MDFU	Yes	31/52 (59.6%)
	No	21/52 (40.4%)
Admitting team for DFD patients	Dedicated MDFU	9/52 (17.3%)
	Vascular Surgery	33/52 (63.5%)
	Orthopaedic surgery	1/52 (1.9%)
	Endocrinology	2/52 (3.8%)
	General medicine	3/52 (5.8%)
	General Surgery	0/52 (0%)
	Other	4/52 (7.7%)
Services provided by MDFU*	Dedicated MDFU ward round	17/31 (54.8%)
	Dedicated MDFU outpatient clinic	30/31 (96.8%)
	Multidisciplinary team meeting	17/31 (54.8%)
	MDFU admission rights	9/31 (29.0%)

*MDFU* Multidisciplinary diabetes foot unit, *DFD* Diabetes-related foot disease

*Among respondents who responded “Yes” to the question “Institution with MDFU”

Of those with available MDFU, all but one institution provided a multidisciplinary outpatient clinic (30/31, 96.8%). A dedicated MDFU ward round was only available in 54.8% of the respondents’ institutions (17/31), most of which were tertiary metropolitan hospitals (14/17, 82.4%).

There was heterogeneity in the admitting teams. Only nine respondents’ MDFU (9/31, 29.0%) functioned as an independent unit with admission rights; while overall 63.5% of patients with DFD requiring hospitalisation were admitted under vascular surgery (33/52).

### Multidisciplinary diabetes foot unit’s composition

**Fig. 1:**
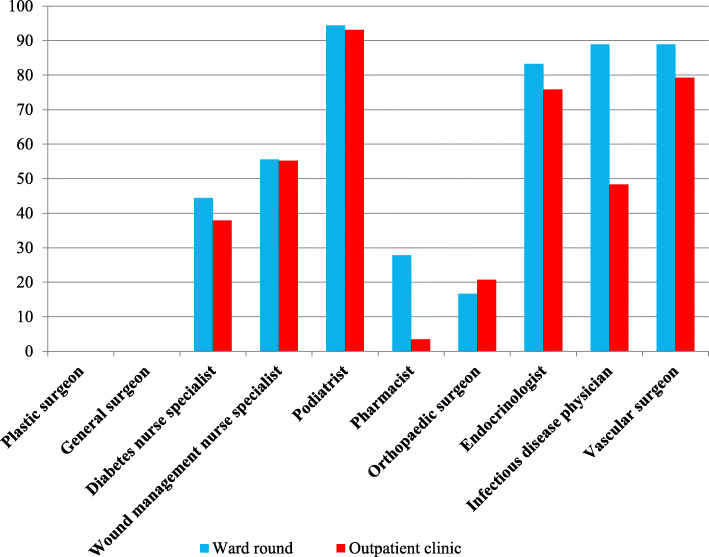
Specialists involved in MDFU ward rounds and outpatient clinics. (MDFU: multidisciplinary diabetes foot unit). The number of responses and respondents answering each question are displayed in table a of Additional File 2

Eighteen respondents responded to further questions regarding composition of their MDFU ward round (Fig. 1). The key members participating in MDFU ward rounds were identified as podiatrists (17/18, 94.4%), vascular surgeons (16/18, 88.9%), infectious disease physicians (16/18, 88.9%), and endocrinologists (15/18, 83.3%). Approximately half of the respondents’ units included a diabetes nurse specialist (8/18, 44.4%) to provide diabetes education, or a wound management nurse specialist (10/18, 55.6%) to optimise wound care.

Twenty-nine responses were received in terms of MDFU composition in the outpatient settings (Fig. 1). The attendance rates of vascular surgeons (23/29, 79.3%), endocrinologists (22/29, 75.9%) and podiatrists (27/29, 93.1%) were similar to those in inpatient settings; while infectious disease physicians were available in less than half of the MDFU outpatient clinics (14/29, 48.3%).

Orthopaedic surgeons were only involved in a much lesser extent (3/18, 16.7% for inpatient; 6/29, 20.7% for outpatient), whilst there was no affiliated plastic, reconstructive or general surgeons at all.

### Outpatient follow-up of DFD patients

Patient follow-up varied according to the degree of intervention and whether an outpatient MDFU clinic was available in each institution.

**Fig. 2:**
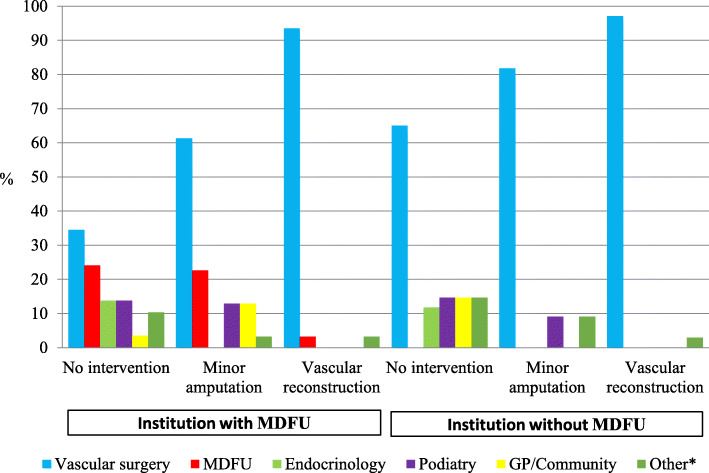
Specialists responsible for follow-up of DFD patients. (DFD: diabetes-related foot disease, MDFU: multidisciplinary diabetes foot unit). The number of responses and respondents answering each question are displayed in table b of Additional File 2. *Follow-up service varied depending by primary admitting team

In institutions without MDFU, the majority of patients were followed up by the vascular surgery service. Rates of vascular follow-up ranged from 65.0% of the patients who did not undergo any intervention (17/34), to 97.1% in those requiring vascular reconstruction (33/34) (Fig. 2 and additional file 2).

In institutions with MDFU, 24.1% (7/29) of patients who required no intervention and 22.6% (7/31) of those with minor amputation would be followed up in MDFU outpatient clinic (Fig. 2 and additional file 2). Vascular surgery was still responsible for a significant proportion of these patient follow-ups with 34.5% (10/29) of those without intervention and 61.3% (19/31) of those with minor amputations being followed up in the vascular surgery clinic. The majority of patients that underwent revascularisation attended dedicated follow-up with vascular surgery (29/31, 93.5%).

Estimated on the survey’s results, endocrinologists, podiatrists, and general practitioners each contributed to approximately 10% of patient follow-up after a hospital admission for DFD.

## Discussion

Although it is widely accepted that a multidisciplinary approach improves outcomes of DFD, at the time of the survey there has been no clear guidance how this care team should be structured. There are no baseline data on how the existing MDFUs are currently functioning. Recently Australian interdisciplinary high risk foot service standards have been introduced and there is a strong interest to establish these throughout the country [17]. This survey helps to establish the status quo of multidisciplinary diabetes foot services in Australia and opens the opportunity for further research in the future to investigate whether the introduction of standards has led to any changes in service provision/ composition of MDFU and more importantly if that leads on to improved outcomes for Australian patients with DFD. The current study suggests four key findings, which are i) gaps between available services in metropolitan vs. rural areas; ii) gaps in service provision between public and private sectors; iii) inconsistent rate of involved specialties; and iv) inconsistent services provision between inpatient and outpatient settings.

The fact that only 60% of the respondents indicated a dedicated MDFU service in their institution with the vast majority being located in metropolitan areas suggests that rural MDFU availability is underdeveloped. In Australia distances are far and long travel times plus cost may prevent DFD patients to seek specialist management early. Furthermore, the Aboriginal and Torres Strait Islander population with its high prevalence of diabetes and high diabetes related complication rates is mainly located in rural parts of the country [20]. This may have a direct influence on the high national major limb amputation rate. An increase in MDFU services outside metropolitan areas where specialist services are available would therefore be desirable.

In Australia privately insured patients have access to a dedicated private hospital system. This survey implies an uneven distribution of multidisciplinary management of DFD between public and private sectors. In fact, all MDFUs identified in this survey were located in public hospitals. By contrast, none of the private hospitals captured in this survey had an established MDFU. The reasons for this are unclear but may reflect challenges in delivery of interdisciplinary care cost-effectively in a private billing environment. Also, the higher DFD prevalence in patients with a lower socio-economic status may lead to diminished demand for such a service in the private sector [21]. However, this finding could mean that privately insured patients with DFD may benefit from direct referrals into the public system regardless of their insurance status.

The National Institute for Health and Care Excellence guidelines published in 2015 in England recommended that a MDFU should consist of specialists in the following areas: diabetology, podiatry, diabetes specialist nursing, vascular surgery, microbiology, orthopaedic surgery, biomechanics and orthoses, and interventional radiology [13]. Notably, less than 20% of MDFUs in this survey reported having a regular orthopaedic surgeon’s input. This is despite the importance of early corrective surgery especially in Charcot’s neuropathy being recognised in several studies as well as guidelines [13, 22, 23]. Therefore, integration of a foot and ankle surgeon into MDFU should be encouraged.

A recent systematic review investigated the impact of MDFU on major limb amputation and identified four key tasks that these units need to address: glycaemic control, local wound management, vascular disease, and infection [12]. This implies that endocrinologists, podiatrists, vascular surgeons and infectious diseases physicians should make the core of such a service. Data from this survey suggests that the majority of Australian MDFU involve these craft groups. However, there is a notable discrepancy between inpatient and outpatient services. The prevalence of MDFU outpatient clinics in our study is similar to the finding of a survey of Australasian infectious diseases clinicians in managing diabetic foot infections [24]. The collected data suggests that the inpatient service is of high quality when available, whilst the outpatient service despite being widely available may still lack adequate access to multidisciplinary expertise. Interestingly, despite the fact that 96% of MDFU in respondents’ institutions offered dedicated MDFU outpatient clinics, the majority of patients were still followed up in vascular surgical outpatient clinics. Whether this is a result of admission practice or a reflection of the lack of specialist availability in the outpatient setting remains unclear.

Despite the availability of inpatient MDFU in more than half of the respondents’ institution in this survey, less than one third of MDFUs had dedicated bed allocations. Inpatients with DFD were admitted under various primary admitting specialties, most commonly vascular surgery. Similar findings were noted in a retrospective audit of patients admitted with DFD in Royal Melbourne Hospital [25]. The benefits of an inpatient MDFU have been widely shown [26] and it also has been demonstrated that multidisciplinary diabetes foot outpatient clinics lead to a reduction in hospital admissions, mortality and cost [19]. Hence the results of this study highlight target areas for improvement of existing models in both inpatient and outpatient settings.

Overall, the survey observed heterogeneity in Australian multidisciplinary care models for DFD. This may have been largely attributed to the lack of coordination and a nationally uniform system to accredit MDFUs. Although several recommendations and statements had been published by peak national bodies [16], they often did not contain detailed strategies to achieve these recommendations and outcome measures to monitor the progress. Germany and Belgium have been on the forefront of standardisation for diabetic foot care since early 2000. Both countries set a world-wide standard by introducing stringent criteria to gain national accreditation as Diabetic Foot Centre [27]. Although this survey was conducted in 2017, it offers a good baseline. Since then, there have been strong efforts to improve MDFU models in Australia. Notably, the National Association of Diabetes Centres published the “Interdisciplinary High Risk Foot Services Standards” in 2018 [17]. These standards allow certification of ‘Interdisciplinary High Risk Diabetes Foot Centres’ and ‘Interdisciplinary High Risk Diabetes Foot Centres of Excellence’. Selection criteria in comparison to the German and Belgian model are displayed in Table 3. It can be expected that the practice is subsequently changing slowly, and the national survey should therefore be repeated in the coming years to monitor and document the progress in delivery of care to patients with DFD.

**Table 3:** Comparison of Belgian, German, and Australian accreditation systems [17, 27]

	Belgium	Germany	Australia
Members	Diabetologist, surgeon on call, podiatrist, diabetes nurse, footwear technician	Diabetologist, at least four of the following: orthopaedic surgeon, vascular surgeon, diabetologist, chiropodist, orthotist, shoemaker, microbiologist	The minimum core staffing is: diabetologist, senior podiatrist, and a credentialled diabetes educator. Patients should have access to vascular surgery and orthopaedic surgery services.
Emergency service	Permanent (24/7) availability of a diabetologist on call	24/7 availability of service	N/A
Outpatient clinic	At least 4 h of consultation per week	N/A	At least one session per week
Dedicated ward round	N/A	N/A	N/A
Evidenced-Based Clinical Management	N/A	Treatment according to guidelines	All members agreed upon treatment guidelines and protocols which are based on published evidence-based best practice guidelines.
Defined intake criteria	N/A	N/A	Evidenced-based intake criteria are clearly defined and articulated to referrers for both urgent and non-urgent referrals.
Coordination	N/A	N/A	A member is appointed as the coordinator to provide overall coordination of the team
Continuity of Care and Communication	Continuity of care during hospitalization Feedback to GPs and home care providers	N/A	Management plans are communicated in a timely manner (within 5 business days) to the referrer and all relevant health professionals involved in the patient’s care including the GP.
Quality improvement	Compulsory audit	Compulsory audit	Compulsory audit

There are several limitations to this study. Firstly, as only non-identifiable and voluntary data was collected multiple responses from the same institutions may have occurred. Thus, the numbers of respondents do not accurately represent the number of institutions. This potentially limits the study’s generalisability. However, vascular surgery units in Australia consist of a relatively small number of consultants. Keeping this in mind in combination with the spread of the number of responses across all Australian states, the authors believe that the study still provides valuable insights into the current service provision for DFD in Australia.

Secondly, being a survey with voluntary response, the study captured only a quarter of practicing vascular surgeons in Australia. However, average response rates to e-mail questionnaires are approximately 25 to 30% [28], so this survey lies within expected levels. The low response rate may reflect the limited numbers of vascular surgeons with an interest in the management of DFD. However, surveying is still considered a methodology for gaining a snapshot of current clinical practice [29].

Lastly, there is a potential selection bias by sending out the survey to a single group of specialists. Vascular surgeons were thought to be a representative group as most patients with DFD would be admitted under vascular surgery or require consultation from vascular surgery. For the purpose of surveying, vascular surgeons can be easily identified and contacted via their professional body; the Australian and New Zealand Society of Vascular Surgery.

## Conclusion

In Australia, the multidisciplinary model for managing DFD was heterogeneous. This study suggests significant differences in MDFU services between the public and private sectors, as well as between outpatient and inpatient settings. Since conducting the survey, new national standards for interdisciplinary high-risk diabetes foot centres have been introduced. A future survey may provide valuable insights into the effect of these standards on MDFU composition and availability.

## Supplementary Information

**Additional file 1.**

**Additional file 2.**

## Data Availability

The datasets used and/or analysed during the current study are available from the corresponding author on reasonable request.

## Abbreviations

DFD: diabetes-related foot disease
MDFU: multidisciplinary diabetes foot unit
IWGDF: International Working Group on the Diabetic Foot
