# Supplementary material for: Availability and service provision of multidisciplinary diabetes foot units in Australia: a cross-sectional survey
Source: J Foot Ankle Res. 2021 Apr 7;14:27. doi: 10.1186/s13047-021-00471-x (PMC8028782; doi:10.1186/s13047-021-00471-x)
Supplement: Supplementary file 1 — Additional file 1. [file 13047_2021_471_MOESM1_ESM.docx]

**Appendix 1: Questionnaire**

1. In which state or territory is your primary location of practice?

- South Australia
- Australian Capital Territory
- New South Wales
- Northern Territory
- Queensland
- Tasmania
- Victoria
- Western Australia

1. In which setting is your primary location of practice?
   - Tertiary metropolitan hospital
   - Secondary metropolitan hospital
   - Regional/rural hospital
   - Private metropolitan hospital
   - Private regional/rural hospital
2. As outpatients, how many diabetic foot patients are seen in your institution per year?
   - <20
   - 21-50
   - 51-100
   - 100+
3. As inpatients, how many diabetic foot patients are seen in your institution per year?
   - <20
   - 21-50
   - 51-100
   - 100+
4. Under which team are the majority of these patient admitted?
   - Dedicated MDFU
   - Vascular surgery
   - Orthopaedic surgery
   - Endocrine
   - General medicine
   - General surgery
   - Other
5. Does your institution have a Multidisciplinary Diabetic Foot Unit (MDFU)?
   - Yes
   - No
6. What is the primary source of referral to your MDFU?
   - ED
   - Inpatient consults
   - Hospital-based outpatient clinic
   - GP
   - Self-referral
   - Private sector
   - Other
7. Which of the following services are included as part of your routine practice? (select all that apply)
   - Ward rounds
   - Outpatient clinic
   - MDT meeting
   - Other
8. Does your institution have a dedicated MDFU ward round?
   - Yes
   - No
9. If yes, which of the following professionals participate in the MDFU ward round? (select all that apply)
   - Vascular surgeon
   - ID physician
   - Endocrinologist/Diabetes Physician
   - Orthopaedic surgeon
   - Pharmacist
   - Podiatrist
   - Wound management nurse specialist
   - Diabetes nurse specialist
   - General surgeon
   - Plastic surgeon
10. Does your institution have a dedicated MDFU outpatient clinic?
    - Yes
    - No
11. If yes, which of the following professionals participate in the MDFU outpatient clinic? (select all that apply)
    - Vascular surgeon
    - ID physician
    - Endocrinologist/Diabetes Physician
    - Orthopaedic surgeon
    - Pharmacist
    - Podiatrist
    - Wound management nurse specialist
    - Diabetes nurse specialist
    - General surgeon
    - Plastic surgeon
12. Does the MDFU in your institution have independent admitting rights?
    - Yes
    - No
13. If not, under which team are diabetic foot patients usually admitted?
    - Vascular surgery
    - Orthopaedic surgery
    - General surgery
    - Plastic surgery
    - Endocrinology
    - General medicine
    - Infectious disease
14. If your institution has MDFU, who is primarily responsible for follow-up of diabetic foot patients who did not have surgery?
    - Vascular surgeon
    - Endocrinologist/Diabetes Physician
    - Podiatry
    - ID physician
    - GP/community
    - MDFU clinic
    - Other
15. If your institution has MDFU, who is primarily responsible for follow-up of diabetic foot patients who had minor amputation/debridement?
    - Vascular surgeon
    - Endocrinologist/Diabetes Physician
    - Podiatry
    - ID physician
    - GP/community
    - MDFU clinic
    - Other
16. If your institution has MDFU, who is primarily responsible for follow-up of diabetic foot patients who had vascular reconstruction?
    - Vascular surgeon
    - Endocrinologist/Diabetes Physician
    - Podiatry
    - ID physician
    - GP/community
    - MDFU clinic
    - Other
17. If your institution does not have MDFU, who is primarily responsible for follow-up of diabetic foot patients did not have surgery?
    - Vascular surgeon
    - Endocrinologist/Diabetes Physician
    - Podiatry
    - ID physician
    - GP/community
    - Other
18. If your institution does not have MDFU, who is primarily responsible for follow-up of diabetic foot patients who had minor amputation/debridement?
    - Vascular surgeon
    - Endocrinologist/Diabetes Physician
    - Podiatry
    - ID physician
    - GP/community
    - Other
19. If your institution does not have MDFU, who is primarily responsible for follow-up of diabetic foot patients had vascular reconstruction?
    - Vascular surgeon
    - Endocrinologist/Diabetes Physician
    - Podiatry
    - ID physician
    - GP/community
    - Other
