# Supplementary material for: Availability and service provision of multidisciplinary diabetes foot units in Australia: a cross-sectional survey
Source: J Foot Ankle Res. 2021 Apr 7;14:27. doi: 10.1186/s13047-021-00471-x (PMC8028782; doi:10.1186/s13047-021-00471-x)
Supplement: Supplementary file 2 — Additional file 2. [file 13047_2021_471_MOESM2_ESM.docx]

**Additional file 2:**

**Table a: Specialists involved in MDFU ward round and outpatient clinic**

|  | **Ward rounds** | **Outpatient clinics** |
| --- | --- | --- |
| Plastic surgeons | 0% (0/18) | 0% (0/29) |
| General surgeons | 0% (0/18) | 0% (0/29) |
| Diabetes nurse specialists | 44.4% (8/18) | 37.9% (11/29) |
| Wound management nurse specialists | 55.6% (10/18) | 55.2% (16/29) |
| Podiatrists | 94.4% (17/18) | 93.1% (27/29) |
| Pharmacists | 27.8% (5/18) | 3.5% (1/29) |
| Orthopaedic surgeons | 16.7% (3/18) | 20.7% (6/29) |
| Endocrinologists | 83.3% (15/18) | 75.9% (22/29) |
| Infectious disease physicians | 88.9% (16/18) | 48.3% (14/29) |
| Vascular surgeons | 88.9% (16/18) | 79.3% (23/29) |

MDFU: multidisciplinary diabetes foot unit

**Table b: Specialists responsible for follow-up of DFD patients**

|  |  | Vascular surgery | MDFU | Endocrinology | Podiatry | GP/Community | Other |
| --- | --- | --- | --- | --- | --- | --- | --- |
| Respondents’ institutions with MDFU | No intervention | 34.5 (10/29) | 24.1 (7/29) | 13.8 (4/29) | 13.8 (4/29) | 3.5 (1/29) | 10.3 (3/29) |
|  | Minor amputation | 61.3 (19/31) | 22.6 (7/31) | 0 (0/31) | 12.9 (4/31) | 0 (0/29) | 3.2 (1/31) |
|  | Vascular reconstruction | 93.5 (29/31) | 3.2 (1/31) | 0 (0/31) | 0 (0/31) | 0 (0/31) | 3.2 (1/31) |
| Respondents’ institutions without MDFU | No intervention | 65.0 (17/34) | 0 (0/34) | 11.8 (4/34) | 14.7 (4/34) | 8.8 (3/34) | 14.7 (5/34) |
|  | Minor amputation | 81.8 (27/33) | 0 (0/33) | 0 (0/33) | 9.1 (3/33) | 0 (0/33) | 9.1 (3/33) |
|  | Vascular reconstruction | 97.1 (33/34) | 0 (0/34) | 0 (0/34) | 0 (0/34) | 0 (0/34) | 2.9 (1/34) |

DFD: diabetes-related foot disease, MDFU: multidisciplinary diabetes foot unit.
